# Association of Serum Total Bilirubin Concentration with Telomere Length: The National Health and Nutrition Examination Survey

**DOI:** 10.1155/2021/4688900

**Published:** 2021-09-23

**Authors:** Lu Hao, Qiuyan Chen, Xi Chen, Qing Zhou

**Affiliations:** ^1^Science and Education Department, Shenzhen Baoan Shiyan People's Hospital, Shenzhen, China; ^2^Central Laboratory, People's Hospital of Baoan District, The Second Affiliated Hospital of Shenzhen University, Shenzhen, China

## Abstract

**Introduction:**

Mildly increased bilirubin concentration has a protective effect on oxidative stress–related diseases. However, it remains unknown whether elevated circulating bilirubin is associated with longer telomere length. The aim of this cross-sectional study was to examine the association between total bilirubin concentration and telomere length.

**Methods:**

We used the data from the National Health and Nutrition Examination Survey (NHANES) 1999-2002. The multivariable linear regression model was used to examine the association between total bilirubin concentration and telomere length. The nonlinear relationship was analyzed using a generalized additive model with the smoothing plot.

**Results:**

A total of 7818 participants with a mean age of 49.20 ± 18.82 years were included. Compared with the lowest concentration of total bilirubin (Q1), the highest quartile of total bilirubin concentration was associated with longer telomere length in male (*β* = 0.04, 95 CI%: 0.00, 0.07, *P* = 0.024) and female (*β* = 0.04, 95 CI%: 0.02, 0.04, *P* = 0.002). Furthermore, an inverted U-shaped relationship between total bilirubin and telomere length was found. On the left of turning points (total bilirubin < 0.5 mg/dL), total bilirubin concentration was positively associated with telomere length (*β* = 0.23, 95 CI%: 0.14, 0.32, *P* < 0.001). However, the association between total bilirubin concentration and telomere length was not significant (*β* = 0.01, 95% CI: -0.01, 0.04, *P* = 0.346) above the turning point.

**Conclusion:**

This is the first evidence based on a nationally representative survey demonstrating a positive and nonlinear association between total bilirubin concentration and telomere length. Future large-scale prospective studies are warranted to confirm our findings.

## 1. Introduction

Telomeres are specialized structures at the ends of eukaryotic chromosomes, and they play a vital role in providing genomic stability and maintaining chromosomal structural integrity [1, 2]. Telomeres consist of repeated and species-specific noncoding DNA sequences and various specifically bound proteins [3]. Because telomeres are gradually shortened in each cell division by semiconservative DNA replication, telomeres are a potential biomarker for cell senescence and apoptosis and age-related diseases [4–6]. Short telomere lengths are associated with all-cause, cardiovascular, and cancer mortality [7, 8]. In addition, leukocyte telomere length may be a cancer prognosis biomarker [9].

Many epidemiologic studies have revealed that telomere length variability and attrition rate are not only determined by genetic background but also affected by environmental and dietary factors [10–12]. Factors that promote telomere shortening include obesity [13], consumption of sugary drinks [14], smoking [15], and decreased physical activity [16]. By contrast, intake of foods with antioxidant and anti-inflammatory properties is associated with longer telomere length [17, 18]. Bilirubin, the end product of heme catabolism in the intravascular compartment, is one of the most potent endogenous antioxidants [19]. For decades, bilirubin has been considered an ominous sign of liver diseases; however, recent evidence suggests that mildly increased bilirubin concentration has a protective effect on oxidative stress–related diseases, including metabolic syndrome [20] and cardiovascular diseases [21]. In addition, a negative correlation between bilirubin and cancer risk has been observed in breast cancer [22], lung cancer [23], and colorectal cancer [24]. High normal levels of total bilirubin are associated with lower cancer mortality [25]. Elevated serum bilirubin levels have also been associated with increased survival in cancer patients [26, 27]. More interestingly, a recent study showed that an occurrence of longer telomeres was observed in male individuals with Gilbert's syndrome chronically exposed to increased unconjugated bilirubin [28]. However, the previous study is limited by retrospective nature and small sample size. It remains unknown whether elevated circulating total bilirubin is associated with longer telomere length among more representative population with a rigorous sampling design.

To fill this knowledge gap, we conducted a cross-sectional study using 1999 to 2002 data from the National Health and Nutrition Examination Survey (NHANES). We examined the association between total bilirubin concentration and telomere length. In addition, on the basis of existing evidence on bilirubin, we examined the nonlinear relation between total bilirubin concentration and telomere length by using a generalized additive model (GAM).

## 2. Materials and Methods

### 2.1. Study Population

The NHANES is designed to assess the health and nutritional status among the noninstitutional civilian United States population and adopted a stratified multistage probabilistic sampling method to select a representative sample in 15 counties across the country. We used NHANES data from 1999 to 2002 [29]. The procedures involving human subject were approved by the National Center for Health Statistics Research Ethics Review Board, and written informed consent was obtained from all participants.

In the NHANES 1999-2002, there were a total of 21004 individuals, and our analysis was limited to 7827 individuals who had data on mean telomere length. Among them, the individuals without total bilirubin concentrations were further excluded (*N* = 9). In the end, a total of 7818 participants aged more than 18 years were included in this cross-sectional study.

### 2.2. Laboratory Analyses

The concentration of total bilirubin was analyzed on Beckman Synchron LX20 by using the timed endpoint Diazo method. In the reaction, bilirubin reacts with diazo reagent in the presence of caffeine, benzoate, and acetate as accelerators to form azobilirubin. The LX20 monitors the change in absorbance at 520 nm at a fixed time interval. This change in absorbance is directly proportional to the concentration of total bilirubin in the sample.

The telomere length assay was performed in the laboratory of Dr. Elizabeth Blackburn at the University of California, San Francisco, using the quantitative polymerase chain reaction (PCR) method to measure telomere length relative to standard reference DNA (T/S ratio), as described in detail previously [30, 31]. Notably, due to the wide variance in telomere length measures across labs and types of assays, the base pair estimates are only comparable to the T/S ratio data generated by the same reference standards and the same laboratory procedures. The conversion from T/S ratio to base pairs was not used, and the primary outcome was mean telomere length (T/S ratio). Each sample was assayed 3 times on 3 days, and the samples were analyzed on duplicate wells, resulting in 6 data points. Each assay plate contained 96 control wells with 8 control DNA samples, which were used to normalize between run variability. The interassay coefficient of variation for telomere length was 6.5%.

### 2.3. Other Variables Analyzed

For all included participants in this study, demographic data on age, sex, race (non-Hispanic white, black, Mexican American, other Hispanic, and other race/ethnicity), and education level (less than high school, high school, and more than high school) were collected. Furthermore, the physical examination and lifestyle factors including body mass index (BMI), smoke status, physical activity, energy intake, alcohol use, diabetes, hypertension, and C-reactive protein were extracted. We categorized BMI as underweight (<18.5), normal (18.5- <25), overweight (25- <30), and obesity (≥30). The smoke status was classified into none, past, and current smoker. Physical activity was estimated by deriving metabolic equivalents for self-reported leisure and normal-time activities and was classified into no aerobic activity, low activity (fewer than 150 min/week), moderate activity (150 to 300 medium intensity min/week), and high activity (4300 medium intensive activity min/week or 4150 high intensity min/week).

### 2.4. Statistical Analysis

The continuous variables were presented as means ± standard deviations. Categorical variables were expressed as the numbers and percentages. The total bilirubin concentrations were categorized based on quintiles (Q1-Q5), and quintile 1 (Q1) was the referent category. The one-way ANOVA (continuous variables) test and chi-square tests (categorical variables) were used to assess the differences between groups. We used univariable and multivariable linear regression model to calculate the *β*-coefficient and 95% confidence intervals (CIs) to evaluate the association between total bilirubin concentration and mean telomere length. The adjusted model was adjusted for age (continuous), race (non-Hispanic white, black, Mexican American, other Hispanic, other race/ethnicity or missing), education (less than high school, high school, more than high school, or missing), body mass index (<18.5, 18.5- <25, 25- <30, ≥30, or missing), smoke status (none, past, current, or missing), alcohol (yes, no, or missing), physical activity (no aerobic activity, low activity, moderate activity, high activity, or missing), energy intake (Q1-Q4), diabetes (yes, no, or missing), and hypertension (yes, no, or missing). We used GAM to identify the nonlinear relationship between total bilirubin concentration and mean telomere length. If the nonlinear relationship was found, the piece-wise linear regression model calculates the threshold effect of total bilirubin concentration and mean telomere length by the smoothing plot. Furthermore, the subgroup analyses were conducted and stratified by age, sex, BMI, smoke status, alcohol, physical activity, energy intake, diabetes, and hypertension. All the statistical analyses were conducted using *R* (http://www.R-project.org) and EmpowerStats software (http://www.empowerstats.com/, X&Y solutions, Inc. Boston MA)). Two-sided *P* values <0.05 were considered as statistical significance.

## 3. Results

The baseline characteristics by quartile of total bilirubin concentration are shown in Table 1. A total of 7818 participants were ultimately included in this cross-sectional analysis. The average age was 49.20 ± 18.82 years, and 51.82% were female. There were 2418 (31.95%) obese participants, 870 (11.23%) diabetes participants, and 2393 (30.77%) hypertension participants. Furthermore, 1694 (21.71%) of the participants were current smokers, and 1952 (26.00%) of the participants have a history of drinking. Participants in the higher quartile of total bilirubin concentration (Q2-Q5) had higher relative telomere length (T/S ratio) compared to those with the lowest total bilirubin concentration (Q1) (*P* = 0.006).

The univariable linear regression for association between total bilirubin concentration and mean telomere length is presented in Table 2. The age, BMI, past smoker, diabetes, hypertension, and C-reactive protein were negatively associated with relative telomere length. In contrast, the female, education, energy intake, physical activity, and alcohol use were positively associated with the telomere length.

Table 3 shows the crude and fully adjusted association between total bilirubin concentrations and mean telomere length by sex. The result of univariate linear regression model showed a significant positive association between total bilirubin concentration and mean telomere length, with *β* of 0.06 (95 CI%: 0.02, 0.09, *P* < 0.001) in male and 0.03 (95CI%: 0.00, 0.06, *P* = 0.048) in female for the fifth vs. the first quartile of total bilirubin concentration. In the multiple linear regression model, compared with the lowest concentration of total bilirubin (Q1), the highest quartile of total bilirubin concentration was associated with increased level of telomere length in male (*β* = 0.04, 95CI%: 0.00, 0.07, *P* = 0.024) and female (*β* = 0.04, 95CI%: 0.02, 0.07, *P* = 0.002). Furthermore, sensitivity analysis results showed that participants tended to have increased level of telomere length as the quintile of serum selenium concentration increased (all *P* for trend <0.01).

**Figure 1:**
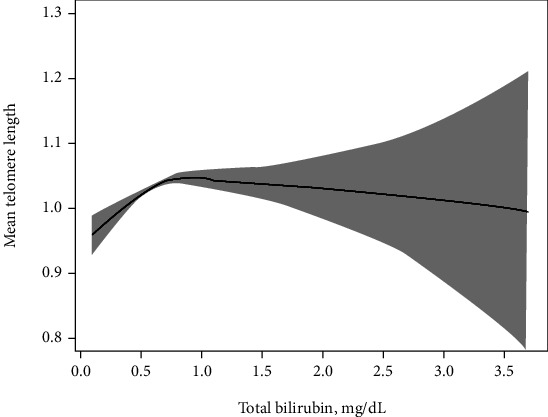
Nonlinear relationship between total bilirubin concentration and telomere length. Adjusted model adjust for age (continuous), race (non-Hispanic white, black, Mexican American, other Hispanic, other race/ethnicity or missing), education (less than high school, high school, more than high school, or missing), body mass index (<18.5, 18.5- <25, 25- <30, ≥30, or missing), smoke status (none, past, current, or missing), alcohol (yes, no, or missing), physical activity (no aerobic activity, low activity, moderate activity, high activity, or missing), energy intake (Q1-Q4), diabetes (yes, no, or missing), hypertension (yes, no, or missing), and C-reactive protein (Q1-Q3).

The GAM model with smoothing curve showed that the total bilirubin concentrations and telomere length were nonlinear after multivariable adjustment (Figure 1). According to the piece-wise linear regression analysis, there were threshold effects between total bilirubin concentrations and telomere length (Table 4), and we calculated that the turning points were 0.5 mg/dL in total bilirubin concentrations. On the left of turning points (total bilirubin < 0.5 mg/dL), we observed a significant positive association between total bilirubin concentration and mean telomere length, with a *β* of 0.23 (95 CI%: 0.14, 0.32, *P* < 0.001). However, the association between total bilirubin concentration and telomere length was not significant (*β* = 0.01, 95% CI: -0.01, 0.04, *P* = 0.346) above the turning point (total bilirubin ≥ 0.5 mg/dL).

Table 5 shows the association between total bilirubin concentration and telomere length in different subgroups. The results showed that the effect sizes of total bilirubin on telomere length were not significant in participants aged ≥60 years. There were no significant interaction effects of BMI, smoke status, energy intake, alcohol use, physical activity, history of diabetes and hypertension, and C-reactive protein.

## 4. Discussion

In this study, we demonstrated a significant positive association between total bilirubin concentration and telomere length in a nationally representative US population. Multivariable regression analysis revealed that higher total bilirubin concentrations (Q1-Q4) were associated with longer telomeres than the lowest total bilirubin concentration. In addition, an inverted U-shaped relationship was observed, indicating that mildly increased bilirubin concentration is protective against telomere shortening. When we added multiple potential confounding factors, including sociodemographics and lifestyle factors and C-reactive protein level, the results were consistent in both crude and fully adjusted models. Sensitivity and stratification analysis of the association between total bilirubin concentration and telomere length was relatively stable and revealed similar results across all subgroups without any indication of interaction. Although unmeasured confounding such as genetic factors and drug therapy may have persisted, the current evidence was sufficiently strong to support robust conclusions.

To our knowledge, our study is the first to indicate a significant positive association between mildly increased total bilirubin concentration and telomere length. Interestingly, similar to our findings, a previous case-control study suggested that Gilbert's syndrome characterized by elevated unconjugated bilirubin in serum was associated with longer telomeres [28]. One possible explanation for this positive association is that unconjugated bilirubin might affect the immune response by downregulating intracellular production of cytokines, as evident from the decreased IL-6 and IL-1*β* level in monocytes from individuals with Gilbert's Syndrome [28]. Previous study has demonstrated that IL-1*β* may be driving telomere attrition due to the faster cell turnover influenced by increased baseline inflammation [32]. Meanwhile, higher expression levels of proinflammatory genes such as *IL-6, IL-1β,* and *IL-8* were associated with shorter telomere length in peripheral blood mononuclear cells [33]. Furthermore, DNA protection was found in the epithelial tissues of older Gilbert's syndrome patients with mildly elevated circulating unconjugated bilirubin [34].

In addition to the widely reported immune response that affects telomere length, increased oxidative stress is believed to play a crucial role in telomere attrition. Animal experiments have demonstrated that chronic oxidative stress can cause telomere shortening in testes, fat, tail, and skin [35]. In addition, high-intensity stress can directly shorten telomere length by inducing telomere double-strand breaks [36]. Human studies have also indicated that dietary and blood-derived antioxidants can delay telomere shortening [37, 38]. Therefore, given the aforementioned direct and indirect evidence, another potential explanation for protective effect of increased total bilirubin concentration on telomere shortening might be attributable to the antioxidant property of bilirubin. Bilirubin belongs to the superfamily of tetrapyrrolic compounds; these compounds can scavenge excess reactive oxygen species, have anti-inflammatory effects, or directly affect cell signaling [39]. Early in vitro studies have indicated that bilirubin is an effective antioxidant under physiological conditions, perhaps having an anti-inflammatory effect [40]. Bilirubin inhibits low-density lipoprotein and lipid oxidation, which can prevent atherosclerotic plaques and, consequently, the development of cardiovascular disease [41]. Observational cohort studies have suggested that a slight increase in bilirubin levels is associated with a reduced risk of cardiovascular disease, supporting the concept of bilirubin as a protective antioxidant [42]. More importantly, bilirubin is the main contributor of the total antioxidant capacity in plasma, and it is more effective than water-soluble antioxidants such as glutathione and vitamin E analogs that are in protecting lipids from oxidation [40, 43].

Notably, our data revealed an inverted U-shaped relationship between total bilirubin concentration and telomere length. Thus, the telomere length increased with increased bilirubin concentration up to 0.5 mg/dL, beyond which this association was not significant. Similar findings were also observed in other observational studies. A large cohort study that included 130 052 patients demonstrated that the association between bilirubin and risk of cardiovascular disease was nonlinear (L shaped). At bilirubin levels <10 to 15 *μ*mol/L, myocardial infarction decreases by 3% to 5% for every 1 *μ*mol/L increase in bilirubin levels [44]. A prospective study revealed a U-shaped relationship between bilirubin concentration and coronary heart disease [45]. Taken together, these results confirm that bilirubin may protect against telomere shortening under normal physiological conditions.

To improve our interpretation of findings and produce more robust results, we adjusted for multiple potential confounding factors, including age, ethnicity, education, BMI, smoking status, alcohol, physical activity, energy intake, diabetes, hypertension, and C-reactive protein. Stratification of the association between total bilirubin concentration and telomere length by sex, BMI, smoking status, energy intake, alcohol use, physical activity, and history of diabetes and hypertension revealed similar results across all subgroups. Meanwhile, our results revealed that the association between total bilirubin and telomere length was not significant in participants aged ≥60 years. Because telomere length is generally considered a reliable biomarker of aging, telomere shortening is associated with a shorter healthy life span [46]. In other words, the older participants in this study may have already had shorter telomeres, leading to the lack of a significant association.

Given telomere length has been investigated as a potential biomarker for cardiovascular disease [47], diabetes mellitus [48], Alzheimer and Parkinson disease [49], chronic obstructive pulmonary disease (COPD) [50], and cancer incidence and mortality [8]. Therefore, identifying the key factors affecting telomere attrition helps prevent and control these chronic diseases and elucidate their pathophysiological mechanisms. Our study's main strength is that it provides the first evidence on the association between total bilirubin concentration and telomere length based on a nationally representative survey. Our findings have critical clinical and public health implications. In addition, we used a GAM with a spline curve to examine the nonlinear relationship between total bilirubin and telomere length. However, this study also has several limitations. First, its cross-sectional design precluded the determination of a causal relationship. Second, the NHANES database does not collect data on unconjugated bilirubin, Gilbert's Syndrome, and cytokines level, such as IL-6 and IL-1*β*. Therefore, we cannot determine the further link between mild unconjugated hyperbilirubinaemia in Gilbert syndrome individuals and increased telomere length, and whether this association is mediated by immune response. However, only limited data are available regarding the association between Gilbert syndrome and telomere length. Future studies using a prospective and large sample size data could further elucidate these issues. Thirdly, although we adjusted carefully for multiple confounders, our findings might still be affected by residual confounders, such as genetic factors and drug therapy. Finally, data on telomere length are limited in the NHANES; large-scale prospective studies are warranted to verify our conclusions.

## 5. Conclusion

In conclusion, our study based on a nationally representative survey demonstrates a positive and nonlinear association between total bilirubin concentration and telomere length. Future large-scale prospective studies are warranted to confirm our findings.

## Figures and Tables

**Table 1:** Characteristics of the participants (*n* = 7818).

Characters	Total bilirubin, mg/dL					
	Q1 (*n* = 1621)	Q2 (*n* = 1210)	Q3 (*n* = 1862)	Q4 (*n* = 1744)	Q5 (*n* = 1381)	*P* value
Age, years	47.20 ± 18.32	49.34 ± 18.83	50.08 ± 18.95	50.55 ± 18.54	49.76 ± 19.37	<0.001
Males, %	428 (26.40)	490 (40.50)	815 (43.77)	1035 (59.35)	999 (72.34)	<0.001
Race, %						<0.001
Non-Hispanic White	701 (43.24)	554 (45.79)	958 (51.45)	954 (54.70)	793 (57.42)	
Non-Hispanic Black	349 (21.53)	237 (19.59)	335 (17.99)	239 (13.70)	171 (12.38)	
Mexican American	421 (25.97)	308 (25.45)	422 (22.66)	422 (24.20)	301 (21.80)	
Other Hispanic	106 (6.54)	66 (5.45)	94 (5.05)	85 (4.87)	66 (4.78)	
Other	44 (2.71)	45 (3.72)	53 (2.85)	44 (2.52)	50 (3.62)	
Education, %						<0.001
< High school	616 (38.12)	458 (37.88)	625 (33.58)	565 (32.45)	371 (26.90)	
High school	367 (22.71)	310 (25.64)	415 (22.30)	380 (21.83)	340 (24.66)	
>High school	633 (39.17)	441 (36.48)	821 (44.12)	796 (45.72)	668 (48.44)	
Body mass index, kg/m^2^						<0.001
<18.5	20 (1.25)	13 (1.11)	24 (1.35)	25 (1.48)	28 (2.10)	
18.5- <25	452 (28.34)	334 (28.43)	476 (26.76)	555 (32.86)	472 (35.46)	
25- <30	506 (31.72)	403 (34.30)	685 (38.50)	631 (37.36)	527 (39.59)	
≥30	617 (38.68)	425 (36.17)	594 (33.39)	478 (28.30)	304 (22.84)	
Cigarette smoker, %						<0.001
None	861 (53.18)	587 (48.55)	945 (50.86)	906 (52.07)	711 (51.63)	
Past	379 (23.41)	329 (27.21)	480 (25.83)	489 (28.10)	422 (30.65)	
Current	379 (23.41)	293 (24.23)	433 (23.30)	345 (19.83)	244 (17.72)	
Physical activity, MET/week						<0.001
No aerobic activity	496 (32.42)	359 (31.55)	497 (28.68)	404 (25.06)	296 (23.31)	
Low activity	423 (27.65)	318 (27.94)	475 (27.41)	425 (26.36)	336 (26.46)	
Moderate activity	247 (16.14)	195 (17.14)	278 (16.04)	303 (18.80)	214 (16.85)	
High activity	364 (23.79)	266 (23.37)	483 (27.87)	480 (29.78)	424 (33.39)	
Energy intake, kcal	2023.20 ± 937.33	2068.62 ± 962.18	2065.69 ± 1029.78	2183.39 ± 1081.76	2223.88 ± 1076.62	<0.001
Alcohol use, %	350 (22.52)	298 (25.56)	373 (20.85)	508 (30.20)	423 (32.12)	<0.001
Diabetes, %	174 (10.83)	178 (14.87)	213 (11.56)	169 (9.77)	136 (9.91)	<0.001
No	1433 (89.17)	1019 (85.13)	1629 (88.44)	1561 (90.23)	1236 (90.09)	
Yes	174 (10.83)	178 (14.87)	213 (11.56)	169 (9.77)	136 (9.91)	
Hypertension, %						0.023
No	1124 (69.38)	800 (66.45)	1261 (67.98)	1214 (70.21)	985 (71.95)	
Yes	496 (30.62)	404 (33.55)	594 (32.02)	515 (29.79)	384 (28.05)	
C-reactive protein						<0.001
Q1	339 (20.91)	334 (27.60)	541 (29.05)	663 (38.02)	671 (48.59)	
Q2	525 (32.39)	391 (32.31)	650 (34.91)	599 (34.35)	462 (33.45)	
Q3	757 (46.70)	485 (40.08)	671 (36.04)	482 (27.64)	248 (17.96)	
Telomere length, T/S ratio	1.01 ± 0.25	1.02 ± 0.26	1.04 ± 0.27	1.03 ± 0.33	1.04 ± 0.27	0.006

**Table 2:** Univariate linear regression for association between total bilirubin concentrations and mean telomere length.

	Number of subjects	Mean telomere length [*β* (95% CI)]	*P* value
Age, years			
<40	2805 (35.88)	Ref	
40- <50	1337 (17.10)	-0.09 (-0.10, -0.07)	<0.001
50- <60	1008 (12.89)	-0.15 (-0.17, -0.13)	<0.001
≥60	2668 (34.13)	-0.25 (-0.26, -0.23)	<0.001
Sex, %			
Male	3767 (48.18)	Ref	
Female	4051 (51.82)	0.04 (0.02, 0.05)	<0.001
Race, %			
Non-Hispanic White	3960 (50.65)	Ref	
Non-Hispanic Black	1331 (17.02)	0.07 (0.05, 0.09)	<0.001
Mexican American	1874 (23.97)	-0.01 (-0.03, 0.00)	0.131
Other Hispanic	417 (5.33)	0.06 (0.03, 0.08)	<0.001
Other	236 (3.02)	0.04 (-0.00, 0.07)	0.055
Education, %			
< High school	2635 (33.76)	Ref	
High school	1812 (23.21)	0.05 (0.03, 0.07)	<0.001
>High school	3359 (43.03)	0.06 (0.05, 0.08)	<0.001
Body mass index, kg/m^2^			
<18.5	2289 (30.24)	0.03 (-0.02, 0.09)	0.229
18.5- <25	110 (1.45)	Ref	
25- <30	2752 (36.36)	-0.04 (-0.05, -0.02)	<0.001
≥30	2418 (31.95)	-0.04 (-0.06, -0.02)	<0.001
Smoke status, %			
None	4010 (51.39)	Ref	
Past	2099 (26.90)	-0.07 (-0.09, -0.06)	<0.001
Current	1694 (21.71)	0.01 (-0.01, 0.03)	0.241
Physical activity, MET/week			
No aerobic activity	2052 (28.18)	Ref	
Low activity	1977 (27.15)	0.04 (0.02, 0.06)	<0.001
Moderate activity	1237 (16.98)	0.04 (0.02, 0.06)	<0.001
High activity	2017 (27.69)	0.05 (0.04, 0.07)	<0.001
Energy intake			
Q1	1697 (22.60)	Ref	
Q2	1787 (23.80)	0.02 (-0.00, 0.03)	0.060
Q3	1833 (24.41)	0.04 (0.02, 0.06)	<0.001
Q4	2191 (29.18)	0.07 (0.06, 0.09)	<0.001
Alcohol use, %			
No	5556 (74.00)	Ref	
Yes	1952 (26.00)	0.02 (0.00, 0.03)	0.015
Diabetes, %			
No	6878 (88.77)	Ref	
Yes	870 (11.23)	-0.08 (-0.10, -0.07)	<0.001
Hypertension, %			
No	5384 (69.23)	Ref	
Yes	2393 (30.77)	-0.08 (-0.09, -0.07)	<0.001
C-reactive protein			
Q1	1056 (13.51)	0	
Q2	3019 (38.62)	-0.08 (-0.10, -0.06)	<0.001
Q3	3743 (47.88)	-0.11 (-0.13, -0.09)	<0.001

**Table 3:** The association between total bilirubin concentrations and mean telomere length by sex.

Total bilirubin, mg/dL	Male (*n* = 3767)		Female (*n* = 4051)		Total (*n* = 7818)	
	*β* (95% CI)	*P* value	*β* (95% CI)	*P* value	*β* (95% CI)	*P* value
Crude model						
Q1	Ref		Ref		Ref	
Q2	0.01 (-0.01, 0.03)	0.567	0.02 (-0.00, 0.04)	0.118	0.02 (-0.00, 0.04)	0.131
Q3	0.03 (0.01, 0.05)	0.112	0.05 (0.03, 0.07)	<0.001	0.04 (0.02, 0.06)	<0.001
Q4	0.04 (0.01, 0.07)	0.017	0.03 (0.00, 0.05)	0.022	0.04 (0.02, 0.05)	<0.001
Q5	0.06 (0.02, 0.09)	<0.001	0.03 (0.00, 0.06)	0.048	0.05 (0.03, 0.07)	<0.001
*P* for trend	<0.001		0.002		<0.001	
Adjusted model^∗^						
Q1	Ref		Ref		Ref	
Q2	0.01 (-0.02, 0.05)	0.457	0.03 (0.01, 0.05)	0.015	0.03 (0.01, 0.04)	0.008
Q3	0.03 (-0.01, 0.06)	0.122	0.06 (0.04, 0.08)	<0.001	0.05 (0.03, 0.07)	<0.001
Q4	0.03 (0.00, 0.06)	0.042	0.05 (0.03, 0.07)	<0.001	0.05 (0.03, 0.06)	<0.001
Q5	0.04 (0.00, 0.07)	0.024	0.04 (0.02, 0.07)	0.002	0.05 (0.03, 0.07)	<0.001
*P* for trend	0.013		<0.001		<0.001	

^∗^Adjusted model adjust for age (continuous), race (non-Hispanic white, black, Mexican American, other Hispanic, other race/ethnicity or missing), education (less than high school, high school, more than high school, or missing), body mass index (<18.5, 18.5- <25, 25- <30, ≥30, or missing), smoke status (none, past, current, or missing), alcohol (yes, no, or missing), physical activity (no aerobic activity, low activity, moderate activity, high activity, or missing), energy intake (Q1-Q4), diabetes (yes, no, or missing), hypertension (yes, no, or missing), and C-reactive protein (Q1-Q3).

**Table 4:** Threshold effect analysis of total bilirubin concentration son mean telomere length using piece-wise linear regression.

	Mean telomere length (*β* (95% CI)) adjusted^∗^	*P* value
Total bilirubin, mg/dL		
<0.5	0.23 (0.14, 0.32)	<0.001
≥0.5	0.01 (-0.01, 0.04)	0.346

^∗^Adjusted model adjust for age (continuous), race (non-Hispanic white, black, Mexican American, other Hispanic, other race/ethnicity or missing), education (less than high school, high school, more than high school, or missing), body mass index (<18.5, 18.5- <25, 25- <30, ≥30, or missing), smoke status (none, past, current, or missing), alcohol (yes, no, or missing), physical activity (no aerobic activity, low activity, moderate activity, high activity, or missing), energy intake (Q1-Q4), diabetes (yes, no, or missing),, hypertension (yes, no, or missing), and C-reactive protein (Q1-Q3).

**Table 5:** The association between total bilirubin concentrations and mean telomere length in different subgroups.

Total bilirubin, mg/dL	Q1 (*n* = 1621)	Q2 (*n* = 1210)		Q3 (*n* = 1862)		Q4 (*n* = 1744)		Q5 (*n* = 1381)	
		*β* (95% CI)	*P* value	*β* (95% CI)	*P* value	*β* (95% CI)	*P* value	*β* (95% CI)	*P* value
Age, year									
<45	Ref	0.04 (0.00, 0.07)	0.026	0.05 (0.02, 0.08)	<0.001	0.04 (0.01, 0.07)	0.006	0.04 (0.01, 0.07)	0.013
45-<60	Ref	0.06 (0.01, 0.10)	0.008	0.08 (0.04, 0.11)	<0.001	0.05 (0.01, 0.08)	0.011	0.05 (0.01, 0.09)	0.007
≥60	Ref	-0.02 (-0.05, 0.00)	0.103	0.00 (-0.02, 0.03)	0.756	-0.00 (-0.03, 0.02)	0.780	-0.01 (-0.03, 0.02)	0.691
Body mass index, kg/m^2^									
<18.5	Ref	0.02 (-0.20, 0.24)	0.885	0.17 (-0.02, 0.37)	0.086	-0.04 (-0.23, 0.15)	0.696	0.01 (-0.19, 0.21)	0.910
18.5- <25	Ref	0.01 (-0.04, 0.05)	0.741	0.06 (0.02, 0.10)	0.003	0.04 (0.00, 0.08)	0.032	0.04 (-0.00, 0.08)	0.078
25- <30	Ref	0.03 (0.00, 0.06)	0.025	0.03 (0.01, 0.06)	0.018	0.04 (0.01, 0.07)	0.007	0.03 (-0.00, 0.06)	0.075
≥30	Ref	0.01 (-0.01, 0.04)	0.337	0.04 (0.02, 0.07)	0.002	0.03 (-0.00, 0.06)	0.064	0.04 (0.01, 0.08)	0.012
Smoke status, %									
None	Ref	0.02 (-0.00, 0.05)	0.097	0.04 (0.02, 0.07)	0.001	0.03 (0.01, 0.06)	0.012	0.02 (-0.01, 0.05)	0.127
Past	Ref	0.02 (-0.01, 0.06)	0.160	0.04 (0.01, 0.07)	0.017	0.05 (0.02, 0.08)	0.003	0.05 (0.02, 0.08)	0.003
Current	Ref	0.02 (-0.02, 0.05)	0.378	0.07 (0.03, 0.10)	<0.001	0.03 (-0.01, 0.07)	0.098	0.06 (0.02, 0.10)	0.005
Energy intake, kcal									
Q1	Ref	0.01 (-0.03, 0.04)	0.687	0.05 (0.01, 0.08)	0.005	0.03 (-0.01, 0.06)	0.155	0.04 (0.00, 0.08)	0.050
Q2	Ref	0.03 (-0.01, 0.06)	0.159	0.03 (-0.00, 0.06)	0.074	0.01 (-0.02, 0.04)	0.546	0.02 (-0.02, 0.06)	0.283
Q3	Ref	0.04 (0.01, 0.08)	0.020	0.06 (0.03, 0.10)	<0.001	0.04 (0.01, 0.08)	0.011	0.03 (-0.00, 0.07)	0.054
Q4	Ref	0.01 (-0.02, 0.05)	0.536	0.05 (0.01, 0.08)	0.005	0.04 (0.01, 0.08)	0.009	0.05 (0.01, 0.08)	0.008
Alcohol use, %									
No	Ref	0.02 (0.00, 0.04)	0.027	0.05 (0.03, 0.07)	<0.001	0.03 (0.01, 0.05)	0.002	0.04 (0.02, 0.06)	<0.001
Yes	Ref	0.01 (-0.02, 0.05)	0.455	0.04 (0.00, 0.07)	0.039	0.03 (-0.00, 0.07)	0.062	0.03 (-0.01, 0.06)	0.156
Physical activity, MET/week									
No aerobic activity	Ref	0.02 (-0.02, 0.06)	0.329	0.04 (0.00, 0.08)	0.032	0.04 (0.00, 0.08)	0.047	0.04 (-0.01, 0.08)	0.094
Low activity	Ref	0.04 (0.01, 0.08)	0.013	0.06 (0.03, 0.09)	<0.001	0.04 (0.01, 0.08)	0.008	0.04 (0.01, 0.08)	0.017
Moderate activity	Ref	-0.00 (-0.05, 0.04)	0.992	0.07 (0.03, 0.11)	0.001	0.04 (0.00, 0.08)	0.049	0.03 (-0.02, 0.07)	0.221
High activity	Ref	0.03 (-0.01, 0.07)	0.092	0.05 (0.02, 0.08)	0.005	0.03 (-0.00, 0.07)	0.076	0.04 (0.00, 0.08)	0.039
Diabetes, %									
No	Ref	0.03 (0.01, 0.05)	0.012	0.05 (0.03, 0.07)	<0.001	0.04 (0.02, 0.06)	<0.001	0.03 (0.01, 0.05)	0.001
Yes	Ref	-0.01 (-0.05, 0.04)	0.782	0.02 (-0.02, 0.07)	0.294	0.03 (-0.02, 0.08)	0.289	0.05 (-0.00, 0.10)	0.065
Hypertension, %									
No	Ref	0.02 (-0.00, 0.05)	0.063	0.05 (0.02, 0.07)	<0.001	0.05 (0.03, 0.07)	<0.001	0.04 (0.02, 0.06)	0.001
Yes	Ref	0.02 (-0.01, 0.05)	0.297	0.05 (0.02, 0.07)	0.001	0.01 (-0.02, 0.04)	0.567	0.03 (0.00, 0.06)	0.043
C-reactive protein									
Q1	Ref	0.03 (-0.01, 0.08)	0.144	0.06 (0.01, 0.10)	0.008	0.04 (-0.00, 0.08)	0.074	0.05 (0.01, 0.09)	0.024
Q2	Ref	0.02 (-0.01, 0.05) 0.1843	0.184	0.04 (0.02, 0.07)	0.002	0.05 (0.02, 0.07)	<0.001	0.02 (-0.01, 0.05)	0.133
Q3	Ref	0.01 (-0.01, 0.04)	0.326	0.04 (0.02, 0.07)	<0.001	0.03 (-0.00, 0.05)	0.059	0.05 (0.01, 0.08)	0.007

^∗^Adjusted model adjust for age (continuous), race (non-Hispanic white, black, Mexican American, other Hispanic, other race/ethnicity or missing), education (less than high school, high school, more than high school, or missing), body mass index (<18.5, 18.5- <25, 25- <30, ≥30, or missing), smoke status (none, past, current, or missing), alcohol (yes, no, or missing), physical activity (no aerobic activity, low activity, moderate activity, high activity, or missing), energy intake (Q1-Q4), diabetes (yes, no, or missing), hypertension (yes, no, or missing), and C-reactive protein (Q1-Q3).

## Data Availability

The data that support the findings of this study are available in the DRYAD repository [Patel, Chirag J. et al. (2016), data from a database of human exposomes and phenomes from the US National Health and Nutrition Examination Survey, Dryad, Dataset, doi:10.5061/dryad.d5h62].
